# Development process in Africa: Poverty, politics and indigenous knowledge

**DOI:** 10.4102/ajod.v3i2.75

**Published:** 2014-06-04

**Authors:** Arne H. Eide, Watson Khupe, Hasheem Mannan

**Affiliations:** 1 SINTEF Technology and Society, Oslo, Norway; 2 Muscular Dystrophy Association, Bulawayo, Zimbabwe; 3 Federation of Organizations of Disabled Persons, Bulawayo, Zimbabwe; 4 Nossal Institute for Global Health, University of Melbourne, Australia

## Abstract

**Background:**

Persons with disability run the danger of not profiting from the development process due to exclusion from basic services and opportunities. Still, the knowledge base on exclusion mechanisms is relatively weak and there is a danger that important aspects are not addressed as they are hidden behind established understandings that are not critically scrutinised.

**Objectives:**

The main purpose of this article was to highlight critical thoughts on prevailing knowledge of the relationship between disability and poverty, the policy base for addressing the rights of persons with disability, and culture as a key component in continued discrimination.

**Method:**

This article aimed at integrating three papers on the above topics presented at the 2011 African Network for Evidence-to-Action on Disability (AfriNEAD) Symposium. The researchers have therefore thoroughly examined and questioned the relationship between disability and poverty, the influence of policy on action, and the role of culture in reproducing injustice.

**Results:**

The article firstly claims that there are limitations in current data collection practice with regards to analysing the relationship between poverty and disability. Secondly, ambitions regarding inclusion of persons with disability in policy processes as well as in implementation of policies are not necessarily implemented in an optimal way. Thirdly, negative aspects of culture in discrimination and bad treatment of disabled need to be highlighted to balance the discussion on disability and culture.

**Conclusion:**

A critical view of prevailing understandings of disability and development is key to producing the knowledge necessary to eradicate poverty amongst persons with disability and other vulnerable groups. Not only do we need research that is actually designed to reveal the mechanisms behind the disability–poverty relationship, we need research that is less tied up with broad political agreements that is not necessarily reflecting the realities at ground level.

## Introduction

According to the World Health Organization, disability affects as many as 15% of the world population (WHO 2011). Whilst this figure is highly uncertain and clearly influenced by the prevailing definition of disability, it is nevertheless an important indication of the magnitude and impact of disability on individuals, families, local communities and societies. The increasing interest in disability in low-income contexts is due amongst other things to the United Nations Convention on the Rights of Persons with Disability (CRPD) (UN 2006) and a growing awareness that disability needs particular attention if the Millennium Development Goals (MDGs) (UN 2000) are to be reached. In order to eradicate discrimination and bring persons with disability into mainstream society, we need to understand the mechanisms that contribute to the disadvantaged situation of persons with disability globally. In this article three perspectives will be discussed in order to illuminate these mechanisms, that is, poverty, politics and culture. The main purpose of the article is to highlight critical thoughts on prevailing knowledge of each of these perspectives in order to contribute to progress in the discourse on disability and development. The article is an integration of three papers presented at the 2011 African Network for Evidence-to-Action on Disability (AfriNEAD) Symposium: the section on disability and poverty is based on the experiences of the first author with regard to disability statistics in poor countries; the section on disability and politics is based on the third author’s experience with regard to studies on disability policy; the section on disability and indigenous knowledge is based on the second author’s experiences as a Zimbabwean activist with disability.

## Disability and poverty

There is currently broad agreement amongst researchers and activists that disability leads to poverty and poverty leads to disability (e.g. Eide & Ingstad 2011; Yeo & Moore 2003). An increasing empirical basis has provided support to the idea of a relationship between disability and poverty, for instance the national surveys undertaken by SINTEF and partners in southern Africa over the last 10 years (Eide *et al*. 2011) and the recent analyses of data from the World Health Survey (Mitra, Posarac & Vick 2012). Common to all such studies of relevance to disability and poverty are, firstly, that they are all cross-sectional, providing evidence of associations but not of causal relationships. Secondly, none of the studies published so far have been designed originally to study the disability–poverty relationship, therefore they are not optimal and have certain validity problems that are not addressed. No data currently exist that for instance could test the complex model presented by Yeo and Moore (2003). This relatively weak empirical basis has also delivered results that should entice critical debate, as results do not always support the disability–poverty relationship; some has yielded results that counter the general assumption, and in many cases demonstrate that the differences between people with disability and people without disability are not always as dramatic as expected (e.g. Loeb *et al*. 2008). Without missing the big picture of obvious discrimination, comparably less access to basic services, and lower standard of living, it may therefore be timely to critically review existing empirical knowledge and call upon research that is properly designed for testing the disability–poverty relationship.

Both disability and poverty are contested concepts that have undergone very important developments over the last few decades. These developments are directly relevant to how the relationship between them is studied.

Firstly, the *International classification of disability, functioning, and health* (WHO 2001) attempts to merge the medical and social models of disability and in effect shifts the balance in WHO’s understanding from the individual and his or her impairment to social participation and environmental barriers. Recent critical analyses (Hughes & Paterson 2010; Shakespeare 2006) have contributed to balancing the medical versus social discourse into an understanding that both perspectives are needed in explaining and analysing the relationship between poverty and disability. A human rights approach to disability has emerged over the last decade or so, with the adoption of the CRPD as a major milestone (UN 2006), strengthening the responsibility at the societal level to avoid any form of discrimination against persons with disability.

Secondly, poverty is increasingly seen as a multidimensional concept (Palmer 2011) and various approaches, such as the basic needs approach, the economic resources approach, and the capability approach, may have different implications for the study of disability and poverty. It is claimed, however, that the prevailing understanding of poverty has moved away from a single measure related to consumption, and instead incorporates a broad spectrum of life domains:

Even the understanding of poverty has broadened from a narrow focus on income and consumption to a multidimensional notion of education, health, social and political participation, personal security and freedom, environmental quality, and so forth. (Wolfensohn & Bourguignon 2004:3)

The factual overlap of these two broadened understandings (of disability and poverty) not only provides a conceptual support for their inter-relationship, but also invites a much *broader* approach to measurement (of disability and poverty) and analyses of the disability–poverty relationship. A human rights approach has clearly penetrated both discourses (on poverty and on disability), and it is interesting to note that the conceptual development implies that the relatively heavy attacks on previously individualised understandings from the disability movement and activists have been effective to a large extent in changing the basis for research on these phenomena (see e.g. Beresford 1996; Coleridge 1993).

As the concepts of poverty and disability have broadened, so have research interests in the field. A range of qualitative studies have presented both thorough descriptions of persons with disability living under poor conditions and more analytical contributions linking individuals’ lived experiences to concepts like social suffering and structural violence (see e.g. Eide & Ingstad 2011). Qualitative studies are able to provide a deeper insight into how disability and poverty plays out together in different contexts, thus contributing very important descriptions and analyses of micro-level mechanisms and how they are directly influenced by social and societal structures. These studies also make it evident that individualisation of disability and poverty has its limitations when the main problems are social or structural.

It is argued that the complexity of disability and poverty requires that research, in order to further scrutinise the disability–poverty relationship, needs to combine perspectives and methods to drive the generation of new knowledge. In combination, different methodological approaches can contribute both to establishing evidence of injustice and differences between groups, and to describing and analysing the reality at ground level, and how individuals live their lives in specific contexts. Whilst qualitative studies are well suited to bringing forward the voices of persons with disability, their perspective and interpretations, disability statistics may contribute to testing relationships between disability and poverty and generate knowledge that can be generalised to larger populations.

## Disability and politics

In Africa, there are a number of key organisations contributing to the progressive realisation of the rights of persons with disabilities. In particular, these actors bring attention to rights of persons with disabilities in the political discourse. The Secretariat of the African Decade of Persons with Disabilities (SADPD) is one of these. The SADPD is engaged in disability advocacy and policy implementation processes by working in partnership with the African Union (AU), governments, civil society and continental, regional and national disabled people’s organisations (DPOs) in Africa. Another leading actor is the Southern Africa Federation of the Disabled (SAFOD), a non-governmental human rights organisation. SAFOD was founded as an umbrella organisation for the national DPOs in the Southern Africa Development Community (SADC). Most significantly, the current United Nations Special Rapporteur, Shuaib Chalklen, from South Africa, continues to make extensive contributions to the advancement of persons with disabilities within both regional and global frameworks. In some of the SADC countries, in particular Angola and South Africa, the disability movement is politically well connected and highly influential, while in other countries DPOs seem to be detached from the country’s political processes.

The United Nations Convention on the Rights of Persons with Disabilities (CRPD) (UN 2006) was adopted by the United Nations General Assembly in 2006, entered into force on 03 May 2008, and to date has been ratified by 132 member states. The CRPD is the first legally binding international instrument with comprehensive protection of the rights of persons with disabilities, and sets out the legal obligations of states to promote and protect the rights of persons with disabilities worldwide. The Committee on the Rights of Persons with Disabilities indicates that the reports of 10 state parties would be due in the course of 2013, bringing the expected total to 69 reports by the end of 2013. However, the report of the Committee on the Rights of Persons with Disabilities (UN 2013c:1) states that ‘at its current pace of work, the Committee is facing a backlog of pending reports that amounts to an eight-year delay between their receipt and examination’.

The fact that persons with disabilities are not included in any of the Millennium Development Goals, Targets or Indicators is likely to be remedied given some recent developments, including the General Assembly resolutions on realising the Goals for persons with disabilities (see resolutions 62/127 (UN 2008a), 63/150 (UN 2008b), 64/131 (UN 2009), 65/186 (UN 2012a), 66/124 (UN 2012b) and 67/140 (UN 2013b)). Most importantly, early in 2013 the High Level Panel on the Post-2015 Development Agenda released *A new global partnership: Eradicate poverty and transform economies through sustainable development* (UN 2013a). This report sets out an agenda to eradicate extreme poverty by 2030, and deliver on the promise of sustainable development with a number of substantive references to persons with disabilities. The report calls for a transformative shift with the mantra ‘leave no one behind’ and names several vulnerable population groups including persons with disabilities. The report calls for indicators that are disaggregated data on disability and stresses that targets should only be considered ‘achieved’ if they are met for all relevant income and social groups. Disability is also represented in goals 1 (eradicating poverty) and 3 (providing quality education and lifelong learning). The report emphasises including persons with disabilities as one of the key stakeholders of strategic relevance along with other excluded groups and institutions in all post-MDG actions and processes. The report recognises that disability forms part of inequality as a cross-cutting issue and advocates the effective and meaningful inclusion of persons with disabilities in all development actions and processes.

This year’s World Health Assembly saw the endorsement of *The action plan for the prevention and control of non-communicable disease* (*NCD*) *2013–2020* (WHO 2013). The development of the action plan follows another important milestone, namely the UN General Assembly convening a high-level meeting in 2011 which brought world leaders together to build awareness and consensus, and which culminated in the adoption of a political declaration on realisation of the MDGs for persons with disability (UN 2012a). In keeping with the tradition of public health its goal is to ‘reduce the preventable and avoidable burden of morbidity, mortality, and disability’ (UN 2012a:4). It calls for member states to:

contribute on a routine basis, information on trends in non-communicable diseases with respect to morbidity, mortality by cause, risk factors and other determinants, disaggregated by age, gender, disability, and socioeconomic groups. (p. 32)

This call for disaggregation by disability is significant given that a vast majority of the leading 20 health conditions associated with disability is non-communicable diseases. The action plan also states that ‘non-communicable disease related to disability (such as amputation, blindness, or paralysis) puts significant demands on social welfare and health systems’ (UN 2012a:2). In addition, it emphasises that ‘rehabilitation needs to be a central health strategy in non-communicable disease programmes’ and seeks access to rehabilitation services to maintain health and functioning (p. 2).

Research evidence of the advancement of the rights of persons with disabilities through policy development and analysis mechanisms within African nations is becoming increasingly available. For example, Schneider *et al*. (2013) analysed 11 African Union (AU) policy documents to ascertain their focus on people with disabilities. The analysis confirmed that these documents provided broad guidelines for individual countries to develop their own national level policies and guidelines. However, very few of them provided implementation plans or monitoring and evaluation guidelines. Also, Mannan *et al*. (2012) evaluated disability and rehabilitation policies of Malawi, Namibia, Sudan and South Africa. The analysis indicated that adequate disability and rehabilitation policies remain mostly undefined, which presents leadership and governance with the opportunity to set this right.

African Policy on Disability and Development (A-PODD) researched the need for disability to be included on the agenda of the Poverty Reduction Strategy Papers (PRSP). The study reported that whilst the PRSP formulation process in Malawi was described as participatory, the disability movement was largely excluded and disability issues were omitted from the document (Wazakili *et al*. 2011a). It also emerged that the disability movement did not participate in the three PRSP formulation processes in Sierra Leone; the first PRSP process is described as a ‘government-donor affair’, the second as ‘more consultative’, and the third as ‘top-down’, and civil society was consulted only to endorse the document, which was based on the ruling party’s manifesto (Wazakili *et al*. 2011b). In Uganda, the study indicated that the lack of utilisation of disability-related research evidence contributed to the exclusion of disability issues from programmes targeting poverty reduction (Chataika *et al*. 2011b). In Ethiopia, the lack of disability-specific data has negatively affected the inclusion of disability issues in socio-economic planning and implementation programmes (Wazakili *et al*. 2011b). Existence of good quality data may increase visibility of disability issues in policy processes, will inform decision and resource allocation, and can be useful for advocacy purposes. These studies thus highlighted greater need to for both utilisation of evidence where available and establishing appropriate disability-specific data in advocacy efforts leading to policy development in Africa.

Whilst disability policy has developed in a very positive direction, with the CRPD and its influence on other international, regional and national policies, and largely incorporating a view of disability as a human rights issue, there are problems with implementation. It is therefore unfortunately the case that the optimism surrounding the CRPD in particular may have a bleak flipside, that is, the celebration of good policies with no effect on the lives of persons with disabilities, in particular in poor contexts where the gap between policy and reality is most pronounced. It is argued that reducing the apparent gap between evidence and policy may alleviate this problem.

## Disability and indigenous knowledge

The literature on indigenous knowledge and disability is mixed. Several authors claim that most indigenous perceptions on disability are positive (Devlieger 2010; Mapara 2009; Ogechi & Ruto 2002). Literature has argued that observed negative practices pertain to poverty and lack of choices rather than negative perceptions of disability. However, most of this literature presents a mixed view (Ingstad 1997; Whyte & Ingstad 1995) in that it also describes negative practices against persons with disability that may be rooted in traditional perceptions and practices. Others, such as Lang and Charowa (2007), have highlighted negative attitudes. In the spirit of this article, which is aimed at presenting a critical view on key aspects of disability and development, and in light of the fact that a balanced view of disability and indigenous knowledge is necessary, this section describes the negative attitudes and practices experienced in Zimbabwe, based on the experiences and reflections of a Zimbabwean disability activist (the second author).

Those who claim that an ethnic group or nation without culture is like a naked person must also concede that at times culture can be used to discriminate against disabled persons. Even during these modern times indigenous people still use culture to justify discrimination against persons with disabilities. In Zimbabwe, cultural reasons are often used successfully as convenient tools to oppress persons with disability and deny their rights. Although there have been nominal positive change as a result of campaigns to stop certain cultural practices that sustain discrimination against disabled persons, some oppressive aspects of culture are still intact. This was confirmed by Marongwe and Mate (2007), as follows:

Sometimes disability is seen as a sign that the women’s ancestors are angry and wish to be appeased. Or, it is attributed to other causes often associated with the baby’s mother’s family or her (immoral) behavior. Men are given to saying that because there are no known Persons with Disabilities (PWDs) in their family the child with disability should not belong to their family. (p. 25)

The rationale here is that nobody can be born with a disability unless there is some witchcraft or divinely generated compound punishment involved.

In most cases, each ethnic group:

has its cultural norms or agreed policy on disabled people within the family or community. But the ultimate result of that norm [*can be*] to confine a disabled person to subhuman status. (Khupe 2010)

For instance, amongst the BaKalanga ethnic group of western Zimbabwe, ‘once a plate or cup has been used by a disabled person it becomes ritually unclean beyond redemption so that no other “normal” human being can use it’ (Khupe 2010). Disability generally was and in many instances still is thought to be spiritually contagious.

Amongst the BaKalanga, when a person with disability dies, the usual burial and post-burial rituals and rites are not performed because it is believed that doing so would bring back the spiritual curse that was attached to that person. All that belonged to him or her is buried with him or her or burnt to ashes. In another example, amongst the Ndebeles in Zimbabwe, a pregnant woman must spit at her tummy whenever she is ‘unfortunate’ to meet a person with disability. Failure to spit on the tummy is thought to cause the woman to give birth to a child with disability. In fact, culturally Ndebele women are discouraged from visiting places where there is a possibility of them meeting a person with disability.

In some traditional contexts, disability is associated with everything that is negative and evil. Such negative cultural embellishment spills over to other spheres of life. According to Lang and Charowa (2007):

disabled people encounter multiple attitudinal, environmental and institutional barriers that militate against their effective inclusion with Zimbabwean society. It is a common perception within Zimbabwe that disabled people are passive and economically unproductive, and therefore constitute a burden upon society. (p. 7)

In many parts of Zimbabwe, many indigenous people believe that the best witch doctor or traditional black magician with the most dangerous traditional medicine, including charms and talismans, must be a person with disability. Ironically, it is culturally agreed by indigenous people that no lucky charms can be prescribed by a disabled *sangoma* or *n’ganga*. Such myths about people with disability have not been easy to dispel, particularly from followers of African traditional religion. According to cultural myths, people with disability are the natural hosts of bad spirits. As such, bad luck or incurable disease must be deposited into the person with disability via different forms of rituals. A common ritual involves having ‘extra-ordinary sex’ with a woman or girl with disability (Khupe 2010). Another ritual involves transferring the bad luck, disease or bad spirit to a person with disability through a gift which would have been ritually imbedded secretly with bad luck or disease that must be handed over directly to the person with disability. Some *sangomas* or *n’gangas* are said to take urine and faeces from a blind woman mixed with some roots to come up with charms that is given to criminals to evade arrest by law enforcement agents. It is believed that such traditional medical concoctions make criminals immune from arrest, hence disability is culturally associated with abnormality and evil.

It is clear that the understanding of disability in traditional communities in Africa, and practices derived from these various understandings, comprise a mix of both ‘positive’ and ‘negative’ patterns, that culture is heterogeneous and changing, and that the cause of negative practices may be found in poverty. However, many forms of abuse of persons with disability perpetrated under the cloak of culture are still prevalent. The more such practices are exposed, the more people move towards their eradication. The CRPD could be a tool for securing basic human rights for persons with disability and putting an end to discriminatory cultural practices.

## Conclusions

The three perspectives discussed above all present critical views on current established knowledge in the disability and development discourse. Firstly, the relationship between disability and poverty is based on limited empirical data. It runs the danger of ‘politically correct’ understanding and opinions undermining the scientific basis in that much of the existing research seems to accept rather uncritically that there is a close relationship. Whilst this very well may be correct, research based on the prevailing understanding as a set precondition will not easily pick up nuances and contradictions and thus run the danger of producing biased results that is not conducive to knowledge production in the long run. This may hamper the contribution of research to support, for instance, the implementation of the CRPD.

Secondly, whilst there are important instruments for implementing policies that support persons with disability and their struggle to access basic services and equal opportunities, it seems clear from the experience with the African Policy and Development study (A-PODD) (Chataika *et al*. 2011a) that these are not sufficient. Even though there are positive developments with regards to policy, the structures that have been responsible for discrimination historically are the very same who will implement the CRPD, and changing discriminatory practices goes deeper that the policy level.

Thirdly, whilst it seems that there is an abundance of culturally and discriminatory practices that cement the exclusion of persons with disability from a normal life, this phenomenon has received limited attention from anthropologists and others within cultural research. It may be argued that much of the cultural research have been blind towards the negative aspects of culture, and at least very limited interest has been shown in revealing such negative practices. If this argument is correct, then this flaw in research may even contribute to upholding the skewed distribution between persons with and without disability in terms of material goods as well as opportunities and access to services in poor countries.

The intention of this article has been strictly to present the views and experiences within a specific African context. Whilst the authors acknowledge the need for a balanced view and for caution with regard to portraying traditional practices in a negative way, it is important to acknowledge that the negative aspects exist alongside the positive aspects found by several authors.

The authors believe that a critical view of prevailing understandings of disability and development is key to producing the knowledge necessary to eradicate poverty, also amongst persons with disability and other vulnerable groups. Not only do we need research that is actually designed to reveal the mechanisms behind the disability–poverty relationship, we need research that is less tied up with broad political agreements that is not necessary reflecting the realities at ground level.
